# Synthesis, Structural Studies and Molecular Modelling of a Novel Imidazoline Derivative with Antifungal Activity

**DOI:** 10.3390/molecules200814761

**Published:** 2015-08-13

**Authors:** Tomasz M. Wróbel, Urszula Kosikowska, Agnieszka A. Kaczor, Sylwia Andrzejczuk, Zbigniew Karczmarzyk, Waldemar Wysocki, Zofia Urbańczyk-Lipkowska, Maja Morawiak, Dariusz Matosiuk

**Affiliations:** 1 Department of Synthesis and Chemical Technology of Pharmaceutical Substances, Medical University of Lublin, Chodzki 4a, 20-093 Lublin, Poland; E-Mails: agnieszka.kaczor@umlub.pl (A.A.K.); dariusz.matosiuk@umlub.pl (D.M.); 2 Department of Pharmaceutical Microbiology, Medical University of Lublin, Chodzki 1, 20-093 Lublin, Poland; E-Mails: urszula.kosikowska@umlub.pl (U.K.); sylwia.andrzejczuk@umlub.pl (S.A.); 3 School of Pharmacy, University of Eastern Finland, Yliopistonranta 1, P.O. Box 1627, FI-70211 Kuopio, Finland; 4 Department of Chemistry, Siedlce University of Natural Sciences and Humanities, 3 Maja 54, 08-110 Siedlce, Poland; E-Mails: kar@uph.edu.pl (Z.K.); wwysocki@uph.edu.pl (W.W.); 5 Institute of Organic Chemistry, Polish Academy of Sciences, Kasprzaka 44/52, 01-224 Warszawa, Poland; E-Mails: zofia.lipkowska@icho.edu.pl (Z.U.-L.); maja.morawiak@icho.edu.pl (M.M.)

**Keywords:** antifungal, *Candida albicans*, X-ray analysis, 14-α-sterol demethylase, molecular modelling, synthesis

## Abstract

Six novel imidazoline derivatives were synthesized and tested in antifungal assays. One of the compounds, *N*-cyclohexyl-2-imino-3-(4-nitrophenyl)imidazolidine-1-carboxamide showed moderate activity against several clinical strains of *Candida albicans*. Its structure was solved by X-ray crystallography and its mode of action was deduced using molecular modelling. It was found to be similar to that of fluconazole. The potential for further optimization including SAR of the compound is briefly discussed.

## 1. Introduction

*Candida* spp. encompasses a class of fungi that includes over 150 species. It exists harmlessly in the body and is a component of the human mycobiome. The mycobiome, which refers to the fungal biota, by interacting with other biomes, as well as with the host, contributes to the progression of fungus-associated diseases and plays an important role in health and disease [1,2]. Fungi are also pathogens that can affect different part of the body. They commonly infect immunocompromised patients, those undergoing chemotherapy, infected with HIV or on immunosuppressive medicines after transplantation [3]. Many serious infections also happen as a result of the development of a fungal biofilm [4]. Moreover fungi cause infections of the skin, hair and nails which are source of substantial discomfort for patients [5,6]. Invasive fungal infections (IFIs) are the most serious type in which brain, heart, blood or other important parts of the body are infected [7,8]. These present the biggest challenge especially in the light of increased occurrence over the past decades and the urgent need for more effective drugs [9]. It is clear that more basic research on new antifungal drugs is needed with emphasis on structural biology, homology modelling and virtual imaging to drive discovery [10].

On the other hand protection of natural microflora, mainly intestinal microbiota should not be neglected. Microorganisms colonizing the human gut are responsible for crucial functions to our health such as elimination of potentially toxic substances or preventing uncontrolled growth of pathogens [11]. It was recently demonstrated that treatment with cyclophosphamide, a drug commonly used in chemotherapy benefits from certain bacteria from the intestinal flora by stimulating fresh immune defense mechanisms [12].

During the course of another investigation [13] we studied novel urea derivatives with antiproliferative properties. Having accounted for their cytotoxicity and consequently the need for protection of the natural microbiological flora we deemed it necessary to check how they influence survival of different microorganisms. We were particularly interested in assessing antifungal activity because of the similarity to imidazole drugs currently in clinical use.

## 2. Results and Discussion

### 2.1. Chemistry

**Scheme 1:**
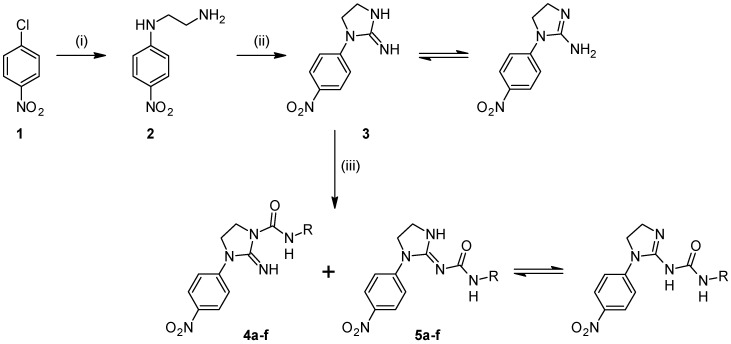
Synthetic sequence. Reagents and conditions: (i) NH_2_CH_2_CH_2_NH_2_, reflux; (ii) 1. BrCN, iPrOH, reflux; 2. NaOH; (iii) RNCO, rt.

**Figure 1:**
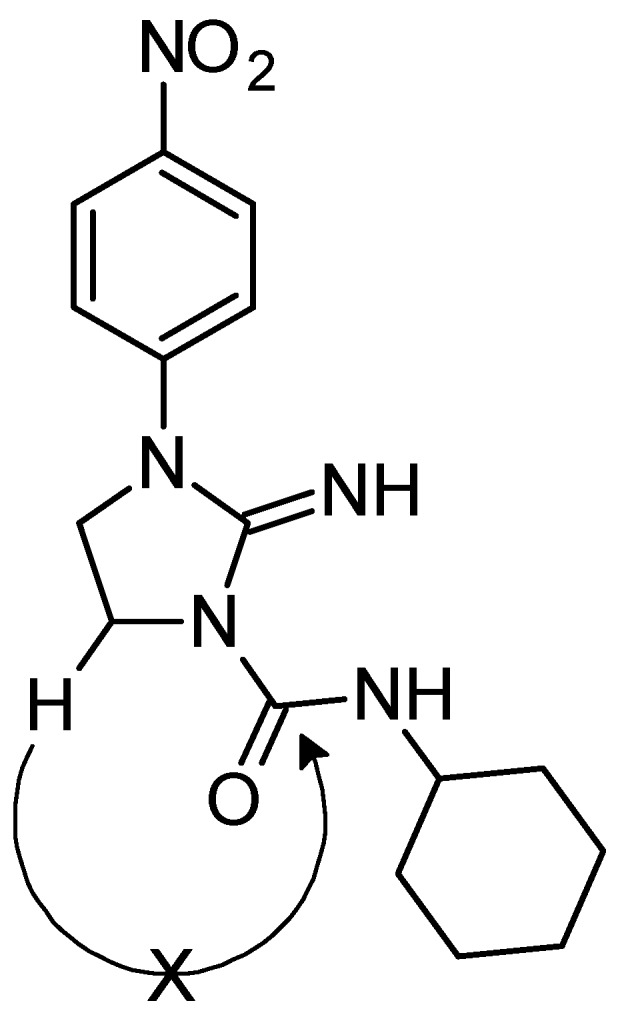
HMBC spectrum of compound **4f** shows absence of expected three-bond correlation between methylene hydrogens H4 and carbonyl carbon C7.

**Figure 2:**
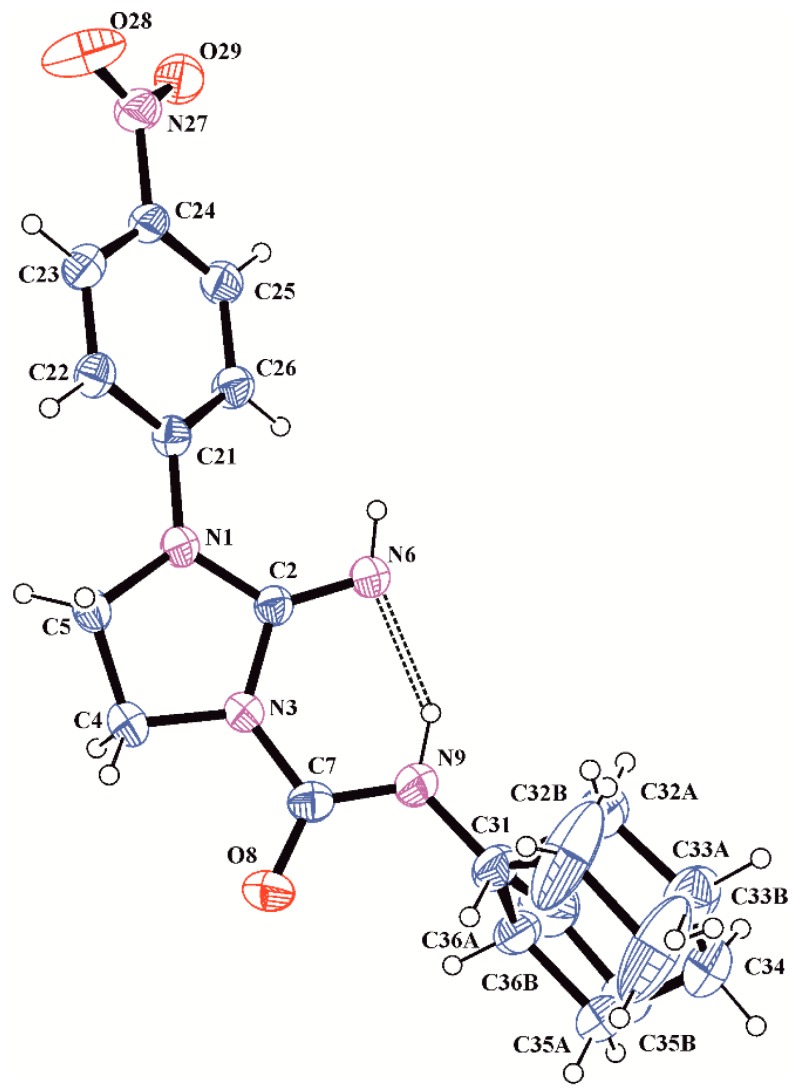
A view of the molecule **4f** with the atom labelling. Non-H atoms are represented by displacement ellipsoids at the 30% probability level.

The compounds presented in this manuscript were synthesized as outlined in Scheme 1. The synthesis started with commercially available 1-chloro-4-nitrobenzene (**1**) which when refluxed with ethylenediamine gave very good yield of *N*^1^-(4-nitrophenyl)ethane-1,2-diamine (**2**). This product was cyclized using cyanogen bromide to afford 1-(4-nitrophenyl)imidazolidin-2-imine (**3**). The last step involved reacting **3** with a variety of isocyanates (Table 1). In most cases a mixture of two regioisomers was obtained, with compounds **4** being the major products. This is probably due to steric hindrance and greater nucleophilicity of the ring nitrogen. This corresponds well with a previous report indicating delocalization of electrons over guanidinium fragment and shortening of the bond between carbon and the ring nitrogen [14]. The obtained regioisomers were virtually indistinguishable by NMR spectroscopy due to their very similar ^1^H-NMR spectra. Additionally, the lack of expected correlation between methylene hydrogens and carbonyl carbon in compounds **4** made this vital clue unavailable. This was confirmed in compound **4f**, whose structure was solved by X-ray crystallography and the measured HMBC spectra did not reveal H4-C7 coupling even using a range of ^3^*J* setting (Figure 1). The position of N-H signals which are most downfield in the ^1^H-NMR spectra may be characteristic for compounds **4**. These signals were shifted beyond or close to 10 ppm, but no consistent pattern was revealed in relation to compounds **5**. However, taking into consideration the fact that these are of poor diagnostic value we relied on X-ray data for absolute certainty (Figure 2). Compounds **5** can exist in two main tautomeric forms, as imidazolidine or as imidazoline derivatives, however X-ray analysis of another reaction product where R = phenyl suggests preference of the former in the solid state [13].

**Table 1:** Isocyanates and substituents in target compounds.

Compound	R
**a**	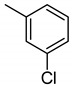
**b**	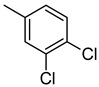
**c**	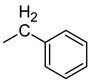
**d**	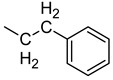
**e**	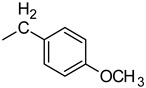
**f**	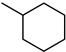

### 2.2. Microbiology

Compound **4f** at 1000 mg/L concentration showed potential activity against some *Candida* species. On the basis of the MIC values it was shown that compound **4f** had strain-dependent activity against both reference strains of *Candida albicans* (MIC = 250–1000 mg/L) and *C. parapsilosis* (MIC = 500 mg/L). Antifungal activity against clinical isolates of *C.* *albicans* was within MIC values ranged from 0.98 to ≥1000 mg/L (Table 2). Clotrimazole used as a reference compound had higher activity against the reference strains (MIC = 1.95–3.9 mg/L) of *Candida* spp. and against clinical isolates of *C. albicans* (MIC = 0.06–7.8 mg/L). The activity assessed on the basis of MIC_50_ = 250 mg/L and MIC_90_ = 1000 mg/L values for **4f** was lower in comparison to clotrimazole activity with MIC_50_ = 0.24 mg/L and MIC_90_ = 1.95 mg/L against *C. albicans* clinical isolates.

**Table 2:** Activity of **4f** and clotrimazole against clinical strains of *Candida albicans* detected on the basis of minimal inhibitory concentration (MIC, mg/L) values.

MIC Value	4f				Clotrimazole			
	No. of Strains (*n* = 41)	% of Strains	MIC_50_	MIC_90_	No. of Strains (*n* = 31)	% of Strains	MIC_50_	MIC_90_
0.015	0 (0.0)	0	250	1000	3	9.7	0.24	1.95
0.03	0	0			5	16.1		
0.06	0	0			2	6.5		
0.12	0	0			3	9.7		
0.24	0	0			5	16.1		
0.49	0	0			5	16.1		
0.98	1	2.4			3	9.7		
1.95	1	2.4			2	6.5		
3.9	1	2.4			1	3.2		
7.82	2	4.9			2	6.5		
15.63	1	2.4			0	0		
31.25	2	4.9			0	0		
62.5	3	7.3			0	0		
125	7	17.1			0	0		
250	5	12.2			0	0		
500	10	24.4			0	0		
1000	7	17.1			0	0		
>1000	1	2.4			0	0		

### 2.3. X-ray Analysis

**Figure 3:**
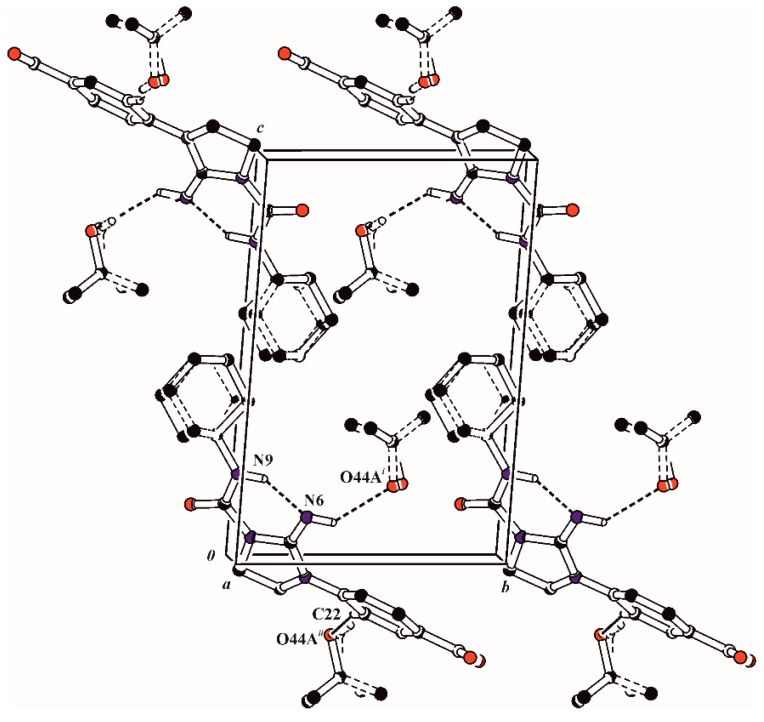
Unit-cell packing of the compound **4f**. H atoms not involved in hydrogen bonding have been excluded.

A detailed X-ray analysis of compound **4f** was performed in order to confirm the synthesis pathway and identification of its tautomeric form in the solid state. Compound **4f** crystallizes as isopropanol solvate with one solvent molecule in the asymmetric unit (Figure 3). The bond lengths and angles are within the normal ranges [15] and are comparable to those found in closely related structures 1-(3-chlorophenyl)-3-(1-*p*-tolylimidazolidin-2-ylidene)urea [16] and 2-(methoxyimino)-*N*-((4-methyl-phenyl)-sulfonyl)imidazolidine-1-carboxamide monohydrate [17]. The bond length of C7=O8, 1.229(3) Å is characteristic of that for the normal C=O double bond (1.230(7) Å in ureas [15]). This value and the positions of the H atoms in the vicinity of the N6 and N9 atoms in the difference electron-density map indicate that compound **4f** exists in the N6-imine/O8-keto tautomeric form in the crystalline state. The guanidine-urea moiety of **4f** is slightly distorted from planarity with maximum deviation from its mean plane of 0.0661(14) Å for the N1 atom.

The conformation of this system is stabilized by an intramolecular N9–H9···N6 hydrogen bond (Table 3). The nitro group in *p*-nitrophenyl substituent is almost coplanar with the benzene ring which is shown by the torsion angle C23–C24–N27–O28 of 11.1(4)^o^, while the benzene and imidazoline rings are twisted of each other with the dihedral angle of 31.79(15)°. The imidazoline ring adopts an envelope conformation, with puckering parameters of Q = 0.252(3) Å and φ = 140.4(7)° [18]. The cyclohexane ring is disordered in two chair conformations A and B (Figure 3), which is confirmed by the puckering parameters of Q = 0.536(9) Å and θ = 8.7(9)° for ring A and Q = 0.57(2) Å and θ = 15.1(2)° for ring B.

**Table 3:** Hydrogen bonding geometry (Å, °) for compound **4f**.

D–H···A	D–H	H···A	D···A	D–H···A	Symmetry Codes
N6–H6···O44A	0.86(4)	2.57(4)	3.263(11)	139(3)	i = x, y, z
C22–H22···O44A	0.93	2.56	3.462(15)	164	ii = 1 − x, 1 − y, −z
N9–H9···N6	0.94(4)	1.88(4)	2.692(3)	143(4)	

**Figure 4:**
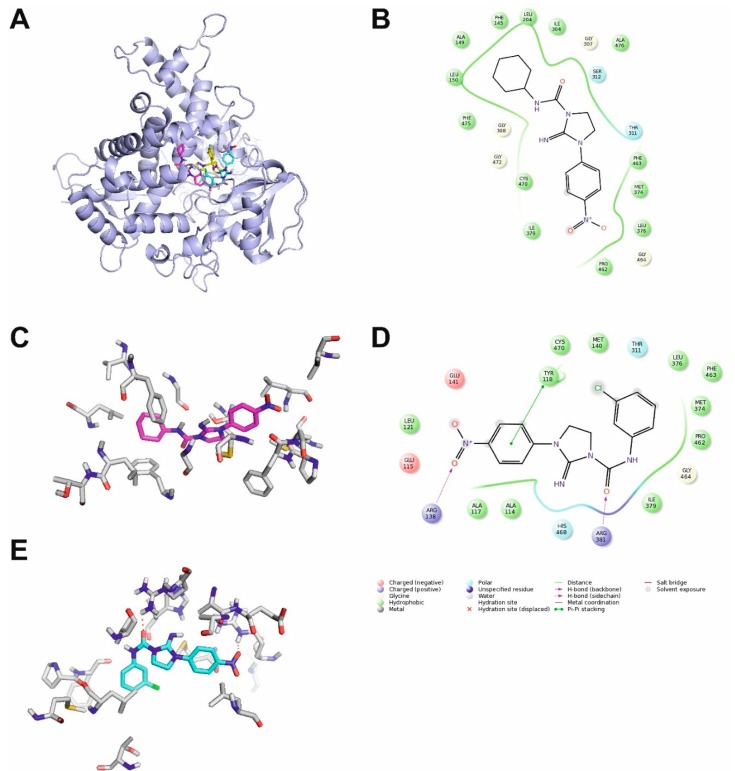
Molecular interactions of **4a** and **4f** with cytochrome P450 14-α-sterol demethylase (homology model) from *Candida albicans*. (**A**) comparison of binding poses of fluconazole (yellow carbon atoms), inactive **4a** (cyan carbon atoms) and active **4f** (magenta carbon atoms). Protein shown in cartoon representation, ligands shown as sticks. Non-polar hydrogen atoms omitted for clarity; (**B**) 2D map of binding site for **4f**; (**C**) details of binding site of **4f**. Ligand and protein shown as sticks with magenta and grey carbon atoms, respectively. Non-polar hydrogen atoms omitted for clarity; (**D**) 2D map of the binding site of **4a**; (**E**) details of binding site of **4a**. Ligand and protein shown as sticks with cyan and grey carbon atoms, respectively. Hydrogen bonds shown as red dashed lines. Non-polar hydrogen atoms omitted for clarity.

The gauche conformation of the cyclohexane ring with respect to the urea moiety is described by the torsion angle C7–N9–C31–C36 of 87.1(5) and 106.3(9)° for rings A and B respectively. In the crystal structure, the molecules of 4f interact with each other by van der Waals forces but in addition each of the these molecules is linked with two molecules of isopropanol solvent via the intermolecular N6–H6···O44A and C22–H22···O44A hydrogen bonds (Table 3, Figure 4).

### 2.4. Molecular Modelling

It is a well-known fact that imidazole derivatives, such as fluconazole exert their pharmacological activity through inhibition of enzyme cytochrome P450 14-α-sterol demethylase from *C. albicans*. Thus, this mechanism of action has already been suggested for similar compounds despite a lack of experimental verification of this hypothesis [19].

As the crystal structure of cytochrome P450 14-α-sterol demethylase from *C. albicans* is not available, the homology model of this enzyme was constructed using cytochrome P450 14-α-sterol demethylase from *Mycobacterium tuberculosis* in complex with fluconazole (PDB ID: 1EA1) [20] as a template with sequence identity 28.97%. The model is similar to earlier published models [21,22,23].

**Figure 5:**
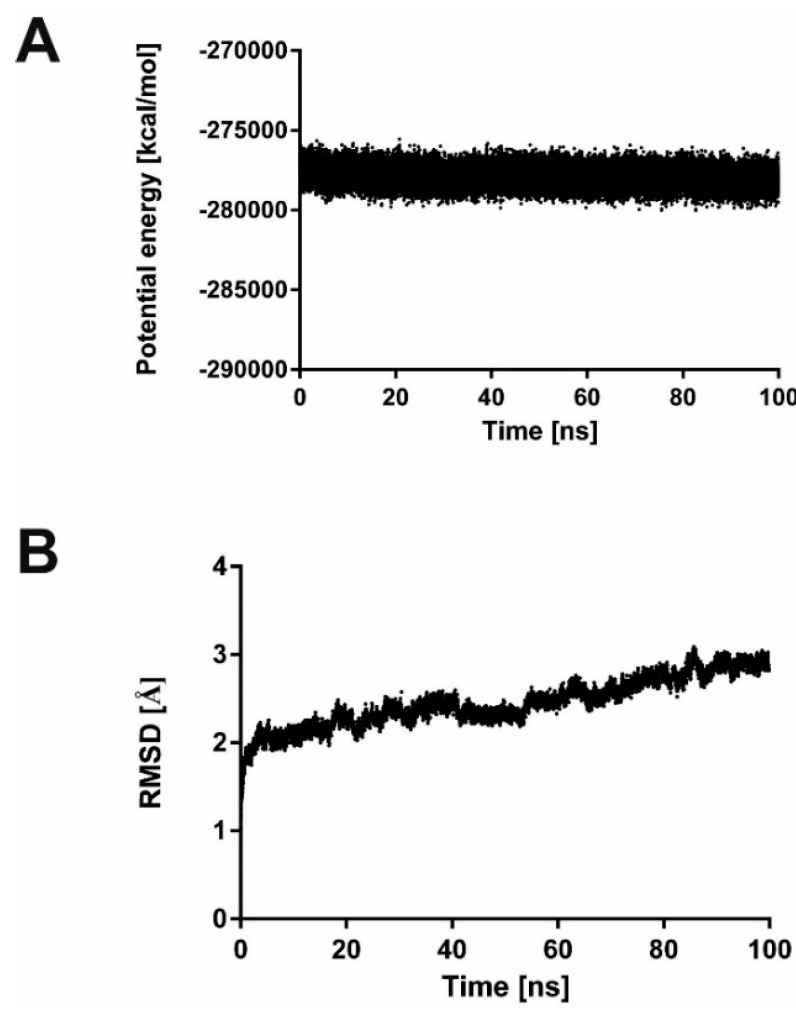

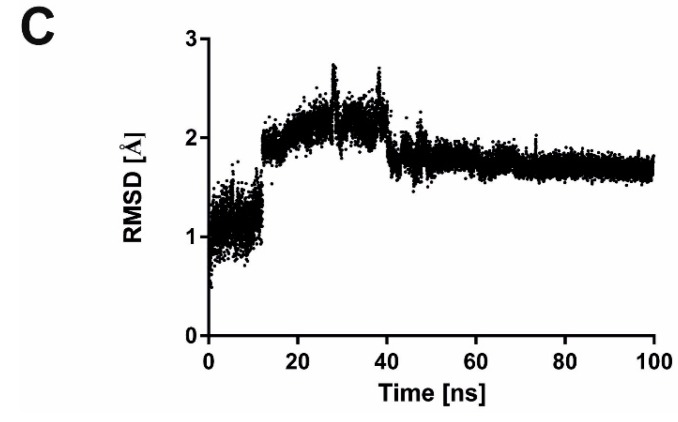
Molecular dynamics simulations results for **4f** in complex with P450 14-α-sterol demethylase (homology model) from *Candida albicans*. (**A**) changes of potential energy for inhibitor-enzyme complex during 100 ns simulations; (**B**) changes in complex RMSD during 100 ns simulations; (**C**) changes in ligand RMSD during 100 ns simulations.

Molecular docking allowed to conclude that the binding pocket occupied by **4f** partially overlaps with the one occupied by fluconazole. The overview of potential inhibitor-enzyme complex is shown in Figure 4A. It can be seen that the position of the active compound **4f** is different than this of the inactive compound **4a**. The details of the binding pocket are presented in Figure 4B,C for **4f** and in Figure 4D,E for **4a**. Similarly for fluconazole in the respective crystal structure of the template, no hydrogen bond interactions were found for **4f** in the binding site of the enzyme. Except for Thr311 and Ser312, only non-polar amino acids are present in the enzyme binding site, including aromatic Phe145, Phe463 and Phe475 as well as aliphatic Ala149, Leu150, Leu204, Ile304, Gly307, Gly308, Cys370, Met374, Leu376, Ile379, Pro462, Gly464, Gly472 and Ala476. The stability of the inhibitor-enzyme complex was confirmed in molecular dynamics simulations. The complex of compound **4f** with cytochrome P450 14-α-sterol demethylase was stable during 100 ns simulations as indicated by decreasing value of complex potential energy (Figure 5A) and complex RMSD (Figure 5B). The stability of selected potential inhibitor binding pose is confirmed by ligand RMSD value below 3 Å Figure 5C). RMSD values were measured in comparison to initial position.

## 3. Experimental Section 

### 3.1. Synthesis

All reagents were purchased from Sigma-Aldrich (St. Louis, MO, USA) and used without further purification unless stated otherwise. NMR spectra were recorded on Fourier 300 and AVANCE III 600 MHz spectrometers (Bruker, Fallanden, Switzerland). Solvents for NMR spectroscopy were obtained from Armar Chemicals (Dottingen, Switzerland). Abbreviations used in NMR spectra s- singlet, d- doublet, t- triplet, q- quarter, m- multiplet, br- broad. MS spectra were acquired on a Bruker microTOF-Q II spectrometer. TLC analysis was carried out on HPTLC Silica gel 60 F_254_ plates (Merck, Darmstadt, Germany) prewashed with methanol.

*N^1^-(4-Nitrophenyl)ethane-1,2-diamine* (**2**). 1-Chloro-4-nitrobenzene (**1**, 10 g, 0.06 mol) and ethylenediamine (40 g, 0.6 mol) were refluxed together for 90 min. Excess ethylenediamine was distilled and the remaining residue was dissolved in hot water, filtered while hot and allowed to cool. Crystals formed very quickly. They were filtered, washed with water and air dried. Thus 9.6 g (83%) of orange solid was obtained. All analytical data corresponds to that found in the literature [24] m.p. 148–150 °C. ^1^H-NMR (300 MHz, DMSO-*d*_6_) δ 7.89–8.07 (m, 2H), 7.30 (br t, *J* = 4.66 Hz, 1H), 6.56–6.72 (m, 2H), 3.13 (q, *J* = 6.08 Hz, 2H), 2.73 (t, *J* = 6.29 Hz, 2H), 1.60 (brs, 2H); ^13^C-NMR (75 MHz, DMSO-*d*_6_) δ 155.2, 135.9, 126.7, 111.1, 46.5, 41.1.

*1-(4-Nitrophenyl)imidazolidin-2-imine* (**3**). Cyanogen bromide (3.18 g, 30 mmol) dissolved in iPrOH (50 mL) was added dropwise to *N*-(4-nitrophenyl)ethane-1,2-diamine (**2**, 5.44 g, 30 mmol) suspended in iPrOH (50 mL). During addition the amine went into solution and colour turned orange-brown. The reaction was heated under reflux for 90 min and then the solvent was removed on a rotavap. The yellow residue was recrystallized from EtOH to afford 7.3 g (85%) of yellow solid as a hydrobromide salt. The salt was converted to a free base form by dissolving it in warm water, adding to 5% NaOH (2.5 eq) and filtering the resulting precipitate. Free base ^1^H-NMR (300 MHz, DMSO-*d*_6_) δ 8.09–8.18 (m, 2H), 7.96–8.07 (m, 2H), 6.06 (brs, 2H), 3.81–3.94 (m, 2H), 3.35–3.43 (m, 2H); ^13^C-NMR (75 MHz, DMSO-*d*_6_) δ 158.5, 148.2, 140.0, 124.9, 117.1, 47.5, 40.0.

#### General Procedure to Obtain Compounds **4**

To a stirred mixture of 1 eq. of 1-(4-nitrophenyl)imidazolidin-2-imine (**3**) in dichloromethane (DCM), tetrahydrofuran (THF) or acetonitrile (ACN) there was added a solution of 1 eq. of an isocyanate in the same solvent. The mixture was stirred for 2 h at room temperature and resulting precipitate filtered (reaction mixture becomes clear as soon as the isocyanate is added and then starts turning cloudy and opaque as it progresses) or the solvent was evaporated.

*N-(3-Chlorophenyl)-2-imino-3-(4-nitrophenyl)imidazolidine-1-carboxamide* (**4a**). THF was used as solvent. The formed precipitate was filtered and washed with THF. ^1^H-NMR (600 MHz, CDCl_3_) δ = 12.28 (brs, 1H), 8.39–8.36 (m, 2H), 7.69 (t, *J* = 2.1 Hz, 1H), 7.41 (ddd, *J* = 0.8, 2.1, 8.2 Hz, 1H), 7.39–7.36 (m, 2H), 7.25 (t, *J* = 8.0 Hz, 1H), 7.06 (ddd, *J* = 0.8, 2.0, 8.0 Hz, 1H), 6.46 (brs, 1H), 4.16–4.11 (m, 2H), 3.99–3.91 (m, 2H); ^13^C-NMR (151 MHz, CDCl_3_) δ = 152.48, 151.24, 144.93, 144.18, 139.59, 134.60, 129.91, 125.81, 123.41, 120.78, 119.67, 117.60, 44.75, 40.66; HRMS (ESI) [M + H]^+^ calc. 360.0858 exp. 360.0855.

*N-(3,4-Dichlorophenyl)-2-imino-3-(4-nitrophenyl)imidazolidine-1-carboxamide* (**4b**). THF was used as solvent. The formed precipitate was filtered and washed with THF. ^1^H-NMR (600 MHz, CDCl_3_) δ = 12.35 (brs, 1H), 8.41–8.36 (m, 2H), 7.81 (d, *J* = 2.6 Hz, 1H), 7.40–7.36 (m, 4H), 6.46 (brs, 1H), 4.15–4.11 (m, 2H), 3.98–3.93 (m, 2H); ^13^C-NMR (151 MHz, CDCl_3_) δ = 152.51, 151.22, 144.86, 144.30, 138.02, 132.72, 130.47, 126.43, 125.87, 121.21, 120.88, 118.87, 44.81, 40.68; HRMS (ESI) [M + H]^+^ calc. 394.0468 exp. 394.0468.

*N-Benzyl-2-imino-3-(4-nitrophenyl)imidazolidine-1-carboxamide* (**4c**). THF was used as solvent and removed on a rotavap when the reaction was finished. The residue was recrystallized repeatedly from iPrOH. ^1^H-NMR (600 MHz, CDCl_3_) δ = 10.10 (brs, 1H), 8.33 (d, *J* = 9.1 Hz, 2H), 7.42–7.24 (m, 7H), 6.30 (s, 1H), 4.57 (d, *J* = 5.8 Hz, 2H), 4.12–4.04 (m, 2H), 3.94–3.85 (m, 2H); ^13^C-NMR (151 MHz, CDCl_3_) δ = 152.48, 151.24, 144.93, 144.18, 139.59, 134.60, 129.91, 125.81, 123.41, 120.78, 119.67, 117.60, 44.75, 40.66; HRMS (ESI) [M + H]^+^ calc. 340.1404 exp. 340.1401.

*2-Imino-3-(4-nitrophenyl)-N-(2-phenylethyl)imidazolidine-1-carboxamide* (**4d**). ACN was used as solvent and removed on a rotavap when the reaction was finished. The residue was recrystallized repeatedly from iPrOH. ^1^H-NMR (600 MHz, CDCl_3_) δ = 9.71 (brs, 1H), 8.37–8.28 (m, 2H), 7.36–7.20 (m, 7H), 6.23 (s, 1H), 4.08–4.00 (m, 2H), 3.91–3.83 (m, 2H), 3.65–3.57 (m, 2H), 2.91 (t, *J* = 7.3 Hz, 2H); ^13^C-NMR (151 MHz, CDCl_3_) δ = 154.04, 152.56, 145.36, 143.75, 139.29, 128.83, 128.48, 126.34, 125.69, 120.40, 44.91, 41.58, 40.72, 36.29; HRMS (ESI) [M + H]^+^ calc. 354.1561 exp. 354.1558.

*2-Imino-N-[(4-methoxyphenyl)methyl]-3-(4-nitrophenyl)imidazolidine-1-carboxamide* (**4e**). DCM was used as solvent and removed on a rotavap when the reaction was finished. The residue was recrystallized repeatedly from iPrOH. ^1^H-NMR (600 MHz, CDCl_3_) δ = 10.01 (brs, 1H), 8.36–8.28 (m, 2H), 7.35–7.29 (m, 4H), 6.93–6.85 (m, 2H), 6.27 (brs, 1H), 4.49 (d, *J* = 5.6 Hz, 2H), 4.09–4.03 (m, 2H), 3.90–3.86 (m, 2H), 3.81 (s, 3H); ^13^C-NMR (151 MHz, CDCl_3_) δ = 158.80, 154.04, 152.58, 145.32, 143.76, 131.11, 128.83, 125.68, 120.40, 113.99, 55.31, 44.92, 43.43, 40.78; HRMS (APCI) [M + H]^+^ calc. 370.1510 exp. 370.1514.

*N-Cyclohexyl-2-imino-3-(4-nitrophenyl)imidazolidine-1-carboxamide* (**4f**). THF was used as solvent. The reaction mixture was filtered through a plug of silica and eluted with fresh THF. The eluant was stripped of solvent and recrystallized repeatedly from iPrOH. ^1^H-NMR (600 MHz, CDCl_3_) δ = 9.64 (br d, *J* = 6.9 Hz, 1H), 8.35–8.31 (m, 2H), 7.33 (d, *J* = 9.0 Hz, 2H), 6.27 (brs, 1H), 4.07–4.02 (m, 2H), 3.90–3.85 (m, 2H), 3.84–3.76 (m, 1H), 2.00–1.91 (m, 2H), 1.76–1.68 (m, 2H), 1.63–1.56 (m, 1H), 1.47–1.38 (m, 2H), 1.38–1.23 (m, 3H); ^13^C-NMR (151 MHz, CDCl_3_) δ = 153.14, 152.73, 145.43, 143.71, 125.68, 120.35, 48.65, 44.90, 40.73, 33.15, 25.71, 24.59; HRMS (ESI) [M + H]^+^ calc. 332.1717 exp. 332.1713.

### 3.2. Microbiology

The antimicrobial activity of six tested compounds was screened using the reference strains of bacteria and fungi. The reference strains of Gram-positive bacteria from American Type Culture Collection (ATCC; LGC Standards, London, UK) included *Staphylococcus aureus* ATCC 25923, *Staphylococcus epidermidis* ATCC 12228, *Bacillus subtilis* ATCC 6633, *Bacillus cereus* ATCC 10876, *Micrococcus luteus* ATCC 10240, while from MICROBANK collection (National Institute of Hygiene—NIH, Warsaw, Poland)—*Staphylococcus aureus* MICROBANK 14001 (MRSA—Methicillin Resistant *Staphylococcus aureus*) were used. The panel of Gram-negative bacteria contained *Escherichia coli* ATCC 25922, *Klebsiella pneumoniae* ATCC 13883, *Proteus mirabilis* ATCC 12453, *Pseudomonas aeruginosa* ATCC 9027. Moreover, the yeasts belonging to *Candida* spp.—3 reference strains (*C. albicans* ATCC 2091, *C. albicans* ATCC 10231, and *C. parapsilosis* ATCC 22019) and 41 clinical isolates of *C. albicans* from the Department of Pharmaceutical Microbiology of Medical University of Lublin collection were used. 

Antimicrobial activity was detected according to the method described previously [25] using microbial suspensions in sterile 0.85% NaCl standardized with an optical density of 0.5 McFarland standard—150 × 10^6^ CFU/mL. All stock solutions of the tested compounds were prepared in DMSO (Sigma-Aldrich). Mueller-Hinton agar buffered at pH 7.2 (for bacteria) or Mueller-Hinton agar supplemented with 2% glucose and buffered at pH 5.6 (for fungi) was used for examination of antibacterial or antifungal activity. Ciprofloxacin and clotrimazole (both from Sigma-Aldrich Co.) were used as reference antibacterial and antifungal agents, respectively.

Initially, the *in vitro* antimicrobial potencies of all tested compounds were screened using the agar dilution method on the basis of the microbial growth inhibition on the agar medium to which the tested compounds were added at concentrations of 1000 mg/L. 10 μL of each microbial suspension was put onto the prepared solid media. The plates were pre-incubated at room temperature for 1–1.5 h and then were incubated at 35 °C for 18 h for bacteria and for 24–48 h for yeasts. The medium with DMSO at the final concentration and without the tested compounds served as a negative control; no microbial growth inhibition was observed.

Subsequently, for the compound **4f** showing an inhibitory effect on the visible growth of some tested bacteria and yeasts, MIC values were estimated using the broth microdilution technique, according to the Clinical and Laboratory Standards Institute (CLSI) standards for bacteria and yeasts, with some modification described previously [25]. The MIC values, defined as the lowest concentration of a compound that prevents visible growth of the tested microorganisms, were determined after 24 h by comparison with the microbial growth in control (compound free) medium.

The microdilution technique was developed using sterile 96-well microplates (Nunc, Roskilde, Denmark), which were inoculated with 2 µL of the microbial suspension (0.5 McFarland standard) put into 198 µL of Mueller Hinton broth without (for bacteria) or with 2% glucose (for fungi) and buffered at pH 7.2 or at pH 5.6, respectively. The two-fold dilution of the tested compound at final concentration from 0.0019 to 1000 mg/L was added to the broth medium. The medium without the tested compound was used as negative control. The optical density (OD_600_) measurements were determined spectrophotometrically (ELx800 microreader, BioTek Instruments, Inc., Winooski, VT, USA) after incubation at 35 °C for 24 h. The blank control wells with two-fold dilution of each of the tested compounds added to broth medium (total volume—200 µL) without the bacteria or yeast suspension were incubated under the same conditions.

### 3.3. X-ray Analysis

Straw-yellow needle-like crystals, suitable for X-ray diffraction analysis, were obtained from an isopropanol solution upon slow evaporation at room temperature. X-ray data of **4f** was collected on a Bruker SMART APEX II CCD diffractometer using CuKα (λ = 1.54178 Å) radiation; crystal sizes 0.26 × 0.25 × 0.18 mm, ω scans, T = 142 K, absorption correction: multi-scan SADABS [26], T_min_/T_max_ = 0.835/0.883. The structure was solved by direct methods using SHELXS-2013/1 [27] and refined by full-matrix least-squares with SHELXL-2014/7 [27]. The C32, C33, C35 and C36 atoms in the cyclohexane ring and the C41, C43 and O44 atoms of the isopropanol molecule were disordered over two positions with a ratio of occupancy of 0.63(1):0.37(1). The N-bond H atoms were located by difference Fourier synthesis and refined freely. The remaining H atoms were positioned geometrically and treated as riding on their parent C atoms with C–H distances of 0.98 Å (methine), 0.97 Å (methylene), 0.96 Å (methyl), 0.93 Å (aromatic) and 0.82 Å (hydroxyl). All H atoms were refined with isotropic displacement parameters taken as 1.5 times those of the respective parent atoms. All calculations were performed using WinGX v. 2014.1 package [28]. CCDC 1412468 contains the supplementary crystallographic data for this paper. These data can be obtained free of charge from The Cambridge Crystallographic Data Centre via www.ccdc.cam.ac.uk.

Crystal data of **4f**: C_16_H_21_N_5_O_3_ C_3_H_7_OH, M = 364.44, triclinic, space group P1, a = 9.0739(3), b = 9.5028(3), c = 14.0775(4) Å, α = 78.537(2), β = 71.537(2), γ = 65.896(2)°, V = 1047.61(6) Å^3^, Z = 2, D_calc_ = 1.241 gcm^−3^, F(000) = 420, μ (CuKα) = 0.727 mm^−1^, T = 142 K, 6787 measured reflections (θ range 5.12–66.58°), 3265 unique reflections (R_int_ = 0.0388) final *R* = 0.0576, *wR* = 0.1772, *S* = 1.063 for 2336 reflections with *I* > 2σ(*I*).

### 3.4. Molecular Modelling

The X-ray structure of cytochrome P450 14-α-sterol demethylase from *Mycobacterium tuberculosis* in complex with fluconazole (PDB ID: 1EA1) [20] was used as a template. Sequence alignment was performed with Muscle software [29]. Homology modelling was carried out using Modeller v.9.14 [30]. A hundred homology models of cytochrome P450 14-α-sterol demethylase from *Candida albicans* in complex with fluconazole were generated, and subsequently assessed by Modeller objective function and Discrete Optimized Protein Energy profiles [31]. The best model was subjected to quality assessments using the Schrödinger software suite [32] tool for Ramachandran plots. Chimera software v. 1.5.3 [33] was used for visualization of results.

Molecular docking was performed using Glide from the Schrödinger software suite [32]. The grid file was generated indicating fluconazole as a reference ligand. The structure of **4a** was modelled using LigPrep protocol from Schrödinger software suite. Crystal structure of **4f** was used as input ligand conformation. Molecular docking of compound **4a** and **4f** was performed using the SP (standard precision) protocol of Glide. 50 poses were generated for the ligand. PyMol v. 0.99 [34] was used for visualization of results.

Molecular dynamics studies of the ligand-receptor complex was performed using Desmond v. 3.0.3.1 [35]. The complex was hydrated and ions were added to neutralize protein charges and then to the concentration of 0.15 M NaCl. The complex was minimized and subjected to MD first in the NVT ensemble for 1 ns and then in NPT ensemble for 20 ns with the restrictions on the protein backbone in each case. The production run was performed in NPT ensemble with no restrictions for 100 ns. Analysis of molecular dynamics simulations was performed with the Schrödinger software suite tools [32].

## 4. Conclusions

Six novel imidazoline derivatives were successfully synthesized and examined for their antifungal activity. All compounds showed no activity in tested assays except compound **4f** which exhibited strain-depending activity against *C. albicans*.

The *Candida* fungi, mainly *C. albicans*, can be opportunistic etiological factors of invasive or non-invasive infections. These yeasts pose the greatest threat especially in immunocompromised and/or hospitalized patients with serious underlying diseases. The search for new compounds active against opportunistic fungi is important because agents available today are characterized by their high toxicity, many side effects, undesired drug interactions, limited routes of administration and very varied activity.

Compound **4f** with antifungal activity against *Candida* spp. therefore possesses potential for further optimization especially since it constitutes a novel structure. Molecular modelling suggested a similar mode of action to that of fluconazole. It can be envisioned that by replacing the saturated imidazoline ring with aromatic imidazole activity could be enhanced. Furthermore the presence of the cyclohexyl substituent is likely to be the key element for the antifungal activity of this compound, as compounds without the cyclohexyl functionality were inactive. Any further modification would need to take into account fact that the majority of amino acids in the binding site are non-polar. This suggests incorporating structural features that lack possibility to form hydrogen bonds.

*Sample Availability*: Samples of the compound 4f are available from the authors.
